# Amyloid Evolution: Antiparallel Replaced by Parallel

**DOI:** 10.1016/j.bpj.2020.03.023

**Published:** 2020-04-07

**Authors:** Ali Asghar Hakami Zanjani, Nicholas P. Reynolds, Afang Zhang, Tanja Schilling, Raffaele Mezzenga, Joshua T. Berryman

**Affiliations:** 1 Department of Physics and Materials Science, University of Luxembourg, Luxembourg City, Luxembourg; 2 ARC Training Centre for Biodevices, Swinburne University of Technology, Melbourne, Victoria, Australia; 3 La Trobe Institute for Molecular Science, Department of Chemistry & Physics, La Trobe University, Bundoora, VIC 3086, Australia; 4 Department of Polymer Materials, Shanghai University, Shanghai, China; 5 Institute of Physics, University of Freiburg, Freiburg im Breisgau, Germany; 6 Departments of Materials; 7 Health Sciences and Technology, ETH Zurich, Zurich, Switzerland

## Abstract

Several atomic structures have now been found for micrometer-scale amyloid fibrils or elongated microcrystals using a range of methods, including NMR, electron microscopy, and X-ray crystallography, with parallel *β*-sheet appearing as the most common secondary structure. The etiology of amyloid disease, however, indicates nanometer-scale assemblies of only tens of peptides as significant agents of cytotoxicity and contagion. By combining solution X-ray with molecular dynamics, we show that antiparallel structure dominates at the first stages of aggregation for a specific set of peptides, being replaced by parallel at large length scales only. This divergence in structure between small and large amyloid aggregates should inform future design of molecular therapeutics against nucleation or intercellular transmission of amyloid. Calculations and an overview from the literature argue that antiparallel order should be the first appearance of structure in many or most amyloid aggregation processes, regardless of the endpoint. Exceptions to this finding should exist, depending inevitably on the sequence and on solution conditions.

## Significance

Probing amyloid formation (and thus amyloid disease) down to the smallest aggregates and earliest timescales, we find that antiparallel *β*-structures have a general thermodynamic advantage at these scales over the parallel structures, which are more commonly observed later and with larger sizes. This has implications for the targeting of therapeutics to limit or redirect early-stage amyloid aggregation.

## Introduction

Toxic amyloid oligomers play a key role in Alzheimer’s, Parkinson’s, and other degenerative diseases (1, 2, 3, 4), whereas functional amyloid can be a valuable and versatile material in nanotechnology and biomedicine (5, 6, 7). To fully understand the process of polymorphic self-assembly, solution structural information is needed, whether obtained by experiment or simulation.

**Figure 1:**
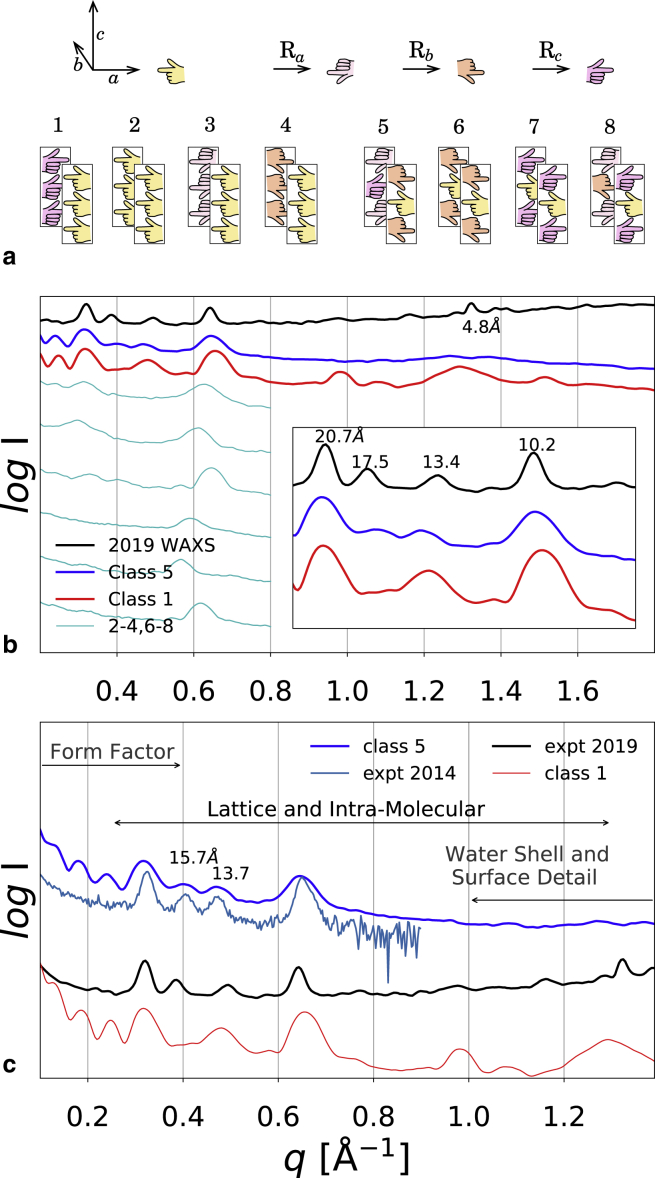
Symmetries and SAXS/WAXS. (*a*) Shown is the construction of test nanocrystal structures obeying each of the eight allowed steric zipper symmetries, which are expected to give eight distinct scattering signatures. (*b*) Shown is the WAXS of the ILQINS peptide solution recorded after 24 h of self-assembly (*black*) and the calculated scattering for MD snapshots of 1296-peptide nanocrystals (6 × 12 × 18) taken at 10 ns (AP 5, *blue*; P 1, *red*). The AP lattice parameters are *a* = 20.6 Å, *b* = 19.1 Å, and *γ* = 82°; the peaks relevant to the lattice shape are annotated with the corresponding Bragg spacing (*main* and *inset*). (*c*) Overlapping regions of the SAXS/WAXS curves are annotated by the type of information that they provide (shape, structure, and solvation). The designed AP structure has extremely good agreement in the structural region with a WAXS curve taken in 2014 and also quite good agreement with these WAXS data. The designed class 1 structure also captures some features of these WAXS data. To see this figure in color, go online.

Amyloids are generally polymorphic at the molecular level, and many examples exist of a given peptide or protein assembling with different morphologies (8,9), including filaments (10), nanotubes (11), helical ribbons (12, 13, 14), twisted ribbons (13,14), and crystals (14,15). Amyloid aggregates formed from the same polypeptide can have different arrangements of the *β*-strands, which have been cataloged as a set of eight “symmetry classes” (16, 17, 18) that are considered as particularly relevant within the larger set of formally available space groups. Fig. 1 shows these symmetry classes diagrammatically, using images of (left) hands. The arrangement of strands within each *β*-sheet is either parallel (P) (classes 1–4) or antiparallel (AP) (classes 5–8); the remaining discrimination between the eight classes is based on side-chain orientation.

Although small length scales and timescales are crucial to understand nucleation events (with single-point mutations often being decisive), the evolution of complex kinetic pathways may also require extremely long times and large lengths to be probed. In addition to being driven by molecular-level differences in contact topology (small length scales and short times), polymorphism may be driven by the buildup of stress in growing mesostructures, causing mechanical transitions to be twisted or buckled aggregate shapes (larger length scales and longer times), which then feed back to the kinetics by altering the assembly-competent surfaces. Polymorphism of this kind has been documented for the model systems studied here (14,19) and also recently observed in reference to ex vivo amyloid-*β* samples taken from patients (20). The importance of complex kinetic pathways and trapping for amyloid aggregation is supported by the rarity with which the global free energy minimum for peptides at finite dilution and physiological temperatures is observed; mesoscopic peptide crystals are rare both in vivo and in vitro despite being the equilibrium destination for aggregation of neutral peptides (21).

In this work, we use a set of hexapeptides that arise as digestion fragments when lysozyme is broken down in a warm acidic environment similar to the stomach as a model of amyloid formers. Hen’s egg-white lysozyme is widely used as a food additive and has close homology to human lysozyme, which is then associated to heritable systemic amyloidosis if it is further mutated. The I_56_LQINS_61_ hexapeptide subsequence of hen’s egg-white lysozyme has been shown in vitro to be a significant driver of aggregation in digested or full-length lysozyme (12) and to have controllable mesostructure polymorphism, with mutations, pH, and initial peptide concentration being used to select in vitro between twisted fibrils (low total aggregation) and rectangular rod-like fibrils or microcrystals (with higher total aggregation) (14), even preserving roughly the same atomistic structure and contact topology. The mutation sequence found to drive increasing aggregation was ILQINS (w.t. chicken) < IFQINS (w.t. human) < TFQINS (disease-associated mutation).

Parallel *β*-sheet atomistic structures for the ILQINS homologs IFQINS and TFQINS have been documented using solid-phase X-ray crystallography (22,23), but prior solution X-ray diffraction from these peptides (14) (and also novel, to our knowledge, higher-resolution X-ray from ILQINS, shown below) generates signals that we argue here are consistent with AP structure in solution, demonstrating atomistic-level polymorphism alongside the mesostructural polymorphism, which has already been discussed. Molecular dynamics (MD) simulations of ILQINS aggregates in differing assembly symmetries are used here to find the atomistic structure most consistent with the results from solution wide-angle X-ray scattering (WAXS) experiments. The most consistent structure (based on calculated WAXS profiles) was AP *β*-sheet conformation, contrary to the crystallography (22,23) and to our earlier proposed oligomer and nanocrystal structure (12). Replica exchange simulations (of unusual scale in terms of the number of peptides treated atomistically) of ILQINS and its homologs confirm that AP *β*-sheets are formed initially for the peptide systems, whereas further atomistic calculations show that P *β*-sheet structures are thermodynamically more favorable only in the limit of larger multisheet assemblies.

We argue here that AP structure has a significant and general thermodynamic advantage relative to P structure at the early stages of aggregation. Similar systems such as the yeast prion fragments GNNQQNY and NNQQNY that have been crystallized in P *β*-sheet (24) have already been the subject of simulation and NMR studies, showing the formation of AP *β*-structures (25, 26, 27, 28, 29) as well as P. The amyloid-*β* (A*β*) (30, 31, 32) and the *α*-synuclein (33) peptides have been shown to form AP (or mixed P/AP) oligomers but parallel fibrils, with important implications for our understanding of neurodegenerative diseases and our strategies for molecular therapy. Only slightly different fibril morphologies may be associated with substantially different diagnoses of amyloid disease (34). Systems of greater amyloidogenicity are found here to show this pattern of AP before P aggregation to a stronger extent than those observed experimentally to have less amyloidogenicity; the diversion toward AP structure does not reduce the ultimate formation of P structure.

The drivers of polymorphism in terms of physical chemistry may include any number of factors, not limited to the pH, concentration, and sequence (already examined by the authors in relation to ILQINS (14,19)) but also including temperature (35), molecular crowding (36), heterogenous nucleation (37), salt (38), electric fields (39), and even stirring or sonication of the reaction vessel (40). In vivo, different cell types have been observed to form tau inclusions of different polymorphism (41), and other forms of amyloid have been observed to be polymorphic across tissue samples from the same patient (42); however, progress toward understanding the physical or biochemical drivers of this variation in the cellular context is limited in the knowledge of the authors to the recent elucidation of atomistic detail for two ex vivo tau polymorphs (34). Looking to the future, we can note that if specific atomistic structures are given, it is feasible in silico to estimate the relative stabilization or destabilization by a given physical effect, particularly the change in electrostatic energies due to pH or salt. Even without atomistic structures, certain effects can be predicted: for example, that backbone hydrogen bonding is typically stronger in AP *β*-sheet (18) such that stabilization of hydrogen bonds, for instance, by reduction of the solvent dielectric, should favor AP structure. In the presence of charge asymmetry about the geometric center of the peptide monomer (this center possibly determined by formation of turns), favoring of AP structure will depend on the pH in relation to the pKa-values of the proton donors and acceptors.

The AP structure may, in some cases, not appear as a kinetic intermediate but as the final observed polymorph. It was hypothesized that antiparallel structure might be finally dominant for fibrils of the (capped) peptide KLVFFAE (A*β*16–22), based on the consistency of candidate AP structures with solid-state NMR results (43), and this would make sense given the strong opposite charges at each end and the symmetric distribution of hydrophobic residues in the center of the sheet. The pattern of strongly metastable antiparallel aggregates is repeated for LVFFA (44) and for microcrystalline amyloid structures of VEALYL, MVGGW, and LYQLEN (16), repeating the features of (approximate) symmetry of hydrophobic residues about the central sequence, with charged residues breaking the symmetry at the termini. The physical origins of the relatively close energetic balance between AP and P *β*-sheets at smaller length scales have been discussed (18,29). An example that we take from the literature to underline the biorelevance of this pattern of initial AP symmetry is the Iowa mutation in the A*β* peptide (D23N-A*β*_1–40_) (45). In the D23N-A*β*_1–40_ example, the symmetry of charge reflected in the geometric center of the peptide chain is reduced by the mutation, stabilizing AP aggregates and causing a heritable disease. The stabilization of AP structure is extremely strong, sufficient to produce observable mature fibril structures; however, these remain metastable with respect to P-*β* amyloid. This is a salient example consistent with a wider pattern in the literature that AP aggregates more often than otherwise have greater tendency to form and greater toxicity than P, but lesser persistence (28, 29, 30, 31, 32, 33).

Analysis of disease-related short-peptide steric zippers has in the past led to the successful design of inhibitors for aggregation of the full-length chain, including aggregation of the A*β* (46) and *τ* (47) peptides. *τ* includes the VQIVYK and VQIINK hexapeptides, which are homologs of ILQINS in terms of hydrophobic and Q/N content. Although the lysine (K) has no cognate in ILQINS, interestingly, in full-length lysozyme, an arginine (R) does immediately follow the serine (S). The effectiveness of inhibitor design was improved by targeting the polymorphic steric zippers for VQIVYK and VQIINK (48), including structural information from soluble nanocrystal or fibril structures, as well as from microcrysytals amenable to solid-phase crystallography. This work indicates that AP polymorphs should be an important target for future inhibitor design and for future design of biomaterials explicitly not templating amyloid formation. This should apply to lysozyme amyloidoses and probably to multiple other amyloid diseases, especially given the examples (1, 2, 3, 4) in which small and often not-yet-imaged peptide assemblies, rather than mesoscopic aggregates, are the toxic species.

## Materials and Methods

### MD and comparison to x ray

Each model crystal had 1296 (6 × 12 × 18) peptides along the *a* (terminus-terminus) × *b* (side chain) × *c* (hydrogen bonding) axes. Each structure was immersed in a periodic box of TIP3P atomistic water (49) and then relaxed for 10 ns by MD simulation at 300 K and 1 atm using the ff14SB atomistic force field (50) and the pmemd software (51). SAXS/WAXS was calculated as an orientationally averaged Fourier transform of the electron density, using CRYSOL (52). CRYSOL parameter choices included the grid order (set as 18), the harmonics cutoff (set as 50), and the number of angular subdivisions (512). Because of the large amount of structural water present in the system, water molecules (and solute hydrogen atoms) were treated explicitly in the calculation. The CRYSOL software was prevented from ignoring water by renaming “WAT” to “NOT” in the input structure file while leaving element names and coordinates intact. To avoid aberrant form factor effects, no imaging was performed on the water such that the solute rested in a diffuse cloud of molecules rather than in a block of explicit water having sharp planar boundaries with the water continuum. The water continuum was set to have a density of 0.334 Å^−3^. Standard SAXS analyses such as radius of gyration were carried out using the ATSAS software (53).

### Energy decomposition

The “docking” free energy is defined with respect to the free energy to bury an interface (a surface perpendicular to the *a*, *b*, or *c* axis). The formula applied to estimate a docking free energy assumes linearity with the number of peptides buried by the interface and independence with respect to the block size in the axis perpendicular to the interface. This assumption should hold approximately true for aggregate sizes above the Bjerrum length. The Bjerrum length is ∼7 Å when measured through water or ∼40 Å when measured through the weaker dielectric of a peptide assembly (assuming *ε*_*r*_ = 15 for a small amyloid oligomer). Beyond the use of nonpolarizable classical force fields, the effective length at which linearity sets in has been found using quantum methods as ∼15 Å (three cells) along the *c* axis (54), implying an even shorter effective Bjerrum length than assumed. Cooperativity (nonlinearity) arises mainly in the hydrogen-bonding direction and is stronger for AP than for P fibrils because of the less-pleated alignment of dipoles, so it should magnify rather than diminish the size of the effect in *ΔG*_*c*_ discussed here. The zipper formation energy *ΔG*_*zip*_ was calculated by splitting a geometry 1 × 2 × 14 into two sheets 1 × 1 × 14. The *c* axis was calculated by splitting a geometry 1 × 1 × 14 in half under the assumption that aggregation in *c* precedes assembly in other directions; thus, the most relevant regime of *ΔG*_*c*_ is single-sheet assembly.

### Hamiltonian replica exchange MD

The HREMD search method (55,56) was implemented using the Nucleic Acid Builder (NAB) molecular manipulation language (57). A generalized Born model (58) was used to represent the solvent as a continuum. Sixty-four replicas of each system were run simultaneously using the developed NAB program and the Amber ff14SB force field (50). The modification to the Hamiltonian for replicas *i* > 0 was to add a harmonic restraint driving each peptide toward an extended reference configuration, with the position and orientation of the reference superimposed on the peptide at each step, but the intrachain degrees of freedom for the reference held constant. The restraint potential was defined as $U\left(\vec{x},\lambda \right)=\left(1/2\right)\lambda {\left|\vec{x}\right|}^{2}$, where $\vec{x}$ is the vector of 3*N* displacements from the atomic coordinates to the corresponding reference coordinates, *λ*_*i*_ = 0.032 (*i*/63)^2^, and the replica index *i* runs over [0..63]. The given functional form for *λ* was verified empirically to provide good mixing between replicas (Fig. S6). The motivation for this was to drive rapid assembly at the high *i* while making transitions down to *i* = 0 such that the unbiased equilibrium conformational ensemble would be sampled (discontinuously) in the *i* = 0 run.

Replica exchanges between adjacent *i*, *j* were attempted at 5-ps intervals and accepted or rejected according to a Metropolis criterion: *w*(*R*_*i*_ ↔ *R*_*j*_) = 1:*ΔU*_*ij*_ ≤ 0 and *w*(*R*_*i*_ ↔ *R*_*j*_) = exp(−*βΔU*_*ij*_):*ΔU*_*ij*_ > 0. The energy change for a candidate exchange event is *ΔU*_*ij*_ = $\left[U\left(\lambda_{i},{\vec{x}}_{j}\right)+U\left(\lambda_{j},{\vec{x}}_{i}\right)\right]-\left[U\left(\lambda_{j},{\vec{x}}_{j}\right)+U\left(\lambda_{i},{\vec{x}}_{i}\right)\right]$. Calculations made use of the University of Luxembourg high-performance computing facility (59).

NAB, pmemd, and ff14SB were all distributed with the AMBER18 software release (60).

### Molecular graphics

Molecular graphics were prepared using PyMOL (61). Secondary structure types for structure images were assigned using DSSP (62). Although DSSP is the acknowledged reference for secondary structure assignment, it has the deficiency that terminus residues are often not assigned *β*-structure despite being strongly ordered; if secondary structure is instead calculated using the heuristic tools in common molecular graphics software, then aggregate images show a larger proportion of *β*-sheet.

### Synthesis and SAXS/WAXS

Synthesis of ILQINS was carried out in solid-phase with *O*-(benzotriazole-1-yl)-1,1,3,3-tetramethylcarbamide tetrafluoroborate as the coupling reagent and *N*,*N*′ diisopropylethylenamine as the base, on Wang Resin (P3 Biosystems, Louisville, KY). 1-Hydroxybenzotriazole was used to prevent unwanted cyclization. The resin was soaked overnight in dimethylformamide (DMF). *O*-(benzotriazole-1-yl)-1,1,3,3-tetramethylcarbamide tetrafluoroborate (four equivalents (equiv)), *N*,*N*′ diisopropylethylenamine (four equiv), Fmoc-protected amino acid (four equiv), and 1-hydroxybenzotriazole (four equiv) in DMF were added and shaken. One hour was allowed for coupling, after which the resin was washed with DMF (4 × 1 min), then DCM (4 × 1 min). The Fmoc protection group was removed with piperidine (15 min). Hydrofluoric acid was used to lift the peptide from the resin (in the presence of 10% anisole at 0°C), allowing 1 h. Tert-butylmethyl ether was used to precipitate the peptide; this was then dissolved in acetic acid and then lyophilized. The lyophilate was then further purified using a reversed-phase high-performance liquid chromatograph with gradients of water and acetonitrile.

The experiment was initiated by mixing dry ILQINS with Milli-Q water to a concentration of 1.5 mM and then allowing it to stand for 24 h. The choice of water without buffering was consistent with previous studies (12,14,19), giving a pH between 6 and 7 depending on the method of measurement (electrodes or chemical indicators). Approximate reference pKa-values of the termini are 8 (NH_3_^+^) and 3 (COO^−^), implying that many or most of the termini retained their charge during assembly.

WAXS was performed at room temperature using the SAXS/WAXS beamline of the Australian Synchrotron (63). Samples were delivered on a robotically controlled *x*, *y* stage holding a 96-well plate and then pumped into the beam through a quartz capillary. Diffraction images were recorded on a PILATUS 1M two-dimensional detector (DECTRIS, Baden-Daettwil, Switzerland); the *q* ranges were between 0.03 and 1.5 Å^−1^. Spectra were recorded with a constant slow flow rate in the capillary (0.15 mL min^−1^) to spread the beam damage. A set of 15 spectra were recorded (exposure time = 1 s), and the average spectrum is shown after background subtraction against Milli-Q water in the same capillary. In the 2014 experiments, a beam of wavelength *λ* = 1.03320 Å (12.0 keV), cross section 300 × 200 *μ*m, and a typical flux of 1.2 × 10^13^ photons per second was used. The 2019 experiments were carried out on the same beamline; however, the x-ray beam was defocused in the vertical direction, spreading the beam over 250 × 500 *μ*m. The use of a wider beam is advanced as a possible partial or complete explanation for the improved signal/noise ratio of the 2019 experiments, as more ILQINS nanostructures were therefore included in the beam area for every spectrum recorded. The peak shifts in the 2019 spectra are not explained by changes in experimental equipment or procedure but might be related to stochastic kinetic effects or to the presence of nucleation seeds ambient in the environment. The two shifted peaks could be directly effected by atomic-level variation, but because they are related to the soft *γ*-degree of freedom, they could also be shifted by coupling to the aggregate mesostructure.

## Results

Comparison between WAXS and calculated WAXS (found using candidate structures allowed to relax using MD) shows that a mixture of P and AP structures are probably present in the solution during the aggregation process. Accelerated simulation techniques were used to discover the initial aggregated states for small systems of 64 peptides; these were found to be dominated decisively by AP structure. Thermodynamic calculations were made, demonstrating that the energetically preferred structure changes from AP to P with increasing aggregate size, a phenomenon that varied quantitatively with sequence change (becoming more pronounced with increased amyloidogenicity) but is seen to arise from general features of the cross-*β* amyloid motif.

### X-ray versus simulation

To make a search for the best three-dimensional ILQINS structure in relation to the WAXS data, one ILQINS model nanocrystal for each of the eight symmetry classes in Fig. 1 *a* was built. All structures were based on the class 1 structure already refined to yield a qualitative match to the X-ray data (12). The new structures in classes 2–8 were subjected to rotations so as to fulfill the appropriate symmetries and also to minor alterations applied by hand so as to reduce clashes and generate favorable contacts (see Fig. S5 for the eight initial structures).

**Figure 2:**
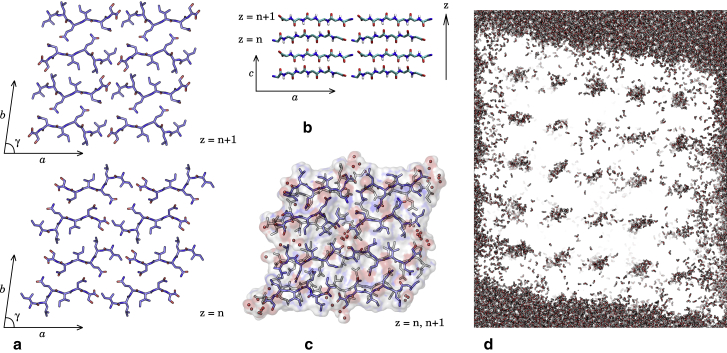
AP structure. (*a*) Two sequential planes are shown perpendicular to the *c* axis; the termini are in close contact on the *a* axis, but not across the steric zipper. (*b*) Given is the plane perpendicular to the *b* axis, showing the backbones of the peptides only. Termini are also in contact along the *c* axis, and hydrogen bonding is strong. (*c*) An MD snapshot of the two planes shown in (*a*) superimposed is given, with semiordered mobile water molecules shown as orange spheres. (*d*) A slice through the whole model aggregate (*c* plane) is given, showing no peptides but only the regular array of water columns. To see this figure in color, go online.

The calculated SAXS/WAXS changed substantially in the course of the simulation for each system, even in class 1, which did not show major structural rearrangement to the naked eye. Among the calculated curves, the structure with symmetry class 5 (Fig. 2 *a*) ended the simulation in a state overall most consistent with this WAXS experiment (Fig. 1), although we should note that the experimental data showed a peak at 4.8 Å, which is a signature of strands in P-*β* alignment but is not seen in the AP *β*-structure because of the 9.7 Å distance on the *c* axis between translated equivalent peptides in this geometry.

Absence of the 4.8 Å reflection in AP amyloid had been confirmed elsewhere via WAXS on AP *α*-synuclein fibrils (38). The partial agreement of both the AP and P structures with this WAXS is contrasted with the good agreement of the AP structure to WAXS taken from an earlier batch of ILQINS peptide under the same protocol (mixed with Milli-Q water and allowed to stand for 24 h) in 2014 (Fig. 2 *c*; data are replotted, having previously appeared in (12)). The two peaks at 15.7 and 13.7 Å are known to be sensitive to small changes in the (mechanically soft) *γ*-angle. Peaks are wider in the simulation than in the experiment because of the smaller size of the model crystallites in relation to the physical aggregates, a constraint imposed by computer hardware limitations. The extreme width of the peak at 1.3 Å^−1^ (∼4.8 Å) in the simulated class 1 system relative to the WAXS data is likely related to the roughly cubic shape of the simulated structures, giving many fewer reflections in the *c* axis than the physical systems, which are highly elongated in *c*.

Fig. 1 *b* (*inset*) and Fig. 1 *c* show that the Bragg reflections between 20 and 10 Å, which are relevant for unit cell geometry, agreed better with the AP structure than with the P structure. The large peaks at ∼20 and ∼10 Å are hypothesized to be related to unit multiples of individual axes (i.e., 100, 200, 010, 020, and 002) and are fitted by a variety of available peptide structures, as the *a* and *b* lattice parameters are roughly constant. The two smaller peaks at 17.5 and ∼13.4 Å are more difficult to assign but have been observed to be sensitive to *γ*, indicating that they involve mixing of two or more nonzero Miller indices; the AP structure class 5 was the only one to stably capture these two peaks. The shapes of calculated and observed SAXS/WAXS profiles diverge at both small and large *q* (outside the range 0.25–0.8 shown in the *inset*). This arises because at small *q*, the form factor of the aggregate dominates the signal, and for the computational systems, this is uniquely defined, whereas for the physical systems, it is an average over a complex distribution, leading to a much smoother curve. At large *q*, the shape of the aggregate again enters in an uncontrolled way, this time by specifying the proportion of different surfaces exposed to the solvent and consequent solvent ordering. In the physical system, scattering from monomers or other small labile species might also be present at high *q*.

Because both of the class 1 and class 5 structures were missing observed peaks, until further refinement can generate a single structure that reproduces the whole of the data, we assume that the real solution contains some proportion of both structures. As we see in Fig. 1, symmetry class 5 has an antiparallel alignment of *β*-strands within *β*-sheets. Fig. 2 shows this structure in detail. A significant aspect of the scattering arises from the entry of water into the structure in regular columns, which took 5–10 ns to reach the center of the lattice when starting from initial conditions of no structural water. All water in the system was included explicitly in the calculations, therefore increasing the computational cost for the orientationally averaged Fourier transform such that forcing or optimizing the dynamical simulation to match the scattering would be impractical, as well as ill-advised given the potential multiplicity of the physical aggregate structures. The 2014 SAXS/WAXS data and atomistic models were deposited in the Small Angle Scattering Biological Data Bank with accession code SASBDB: SASDHQ5, and the 2019 data were deposited with code SASBDB: SASDHK8.

The structure arrived at by comparing WAXS to unconstrained and unguided MD does not have the status of 1-Å-resolution crystallography outputs. The limited information from solution scattering compared to crystallography means that although a match of calculated to observed scattering is information in favor of a given structure being correct, further computational analysis (carried out below) is needed to increase the credibility of the candidate structures and to provide kinetic-thermodynamic explanations of why and in which circumstances they should be formed.

The small-angle section of the SAXS/WAXS curve can in theory be interpreted to provide a cross-sectional radius of gyration; however, this analysis is not accurate for polydisperse samples. Understanding that the sample is polydisperse, for completeness, we report values of 19.74 Å from the 2014 data and 24.8 Å from the 2019 data, which are consistent with the small-angle part of the data reporting primarily on single- or face-to-face-paired *β*-sheets (without lateral unit cells, therefore not giving scattering in the medium-q region). It is tempting to link the thicker radius of gyration and increased 4.8 Å peak of the 2019 data to the presence of more paired parallel *β*-sheet steric zippers in this later experiment, which agrees less well with the AP computational model structures; however, we are not completely confident in this analysis because of the problem of polydispersity. If this analysis is correct, then it is entirely consistent with the thermodynamic calculations presented below.

### Simulated aggregation of 64 peptide systems

**Figure 3:**
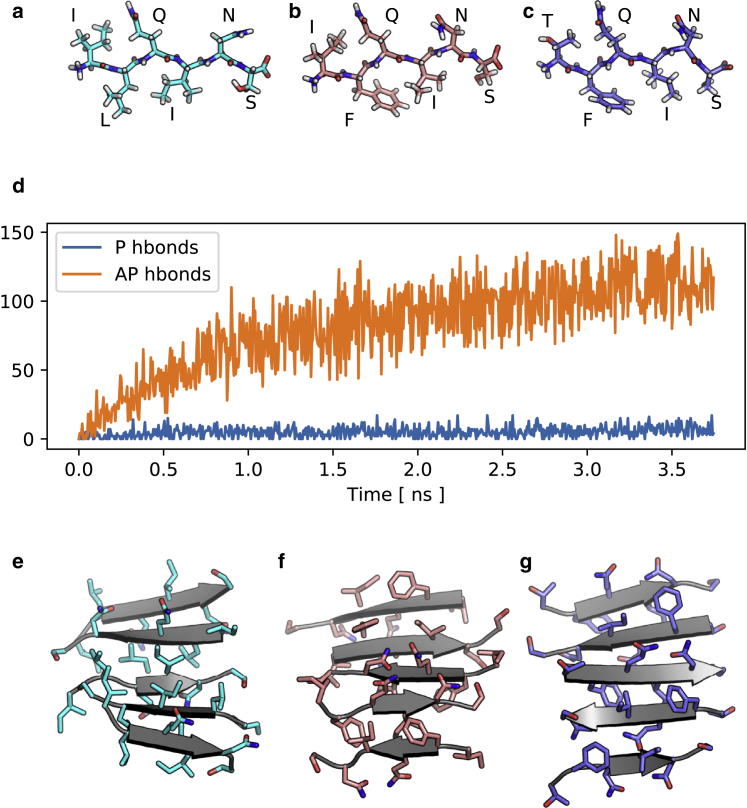
Replica exchange. (*a*–*c*) Shown are the extended reference peptides used for HREMD simulations; these peptides have a pleated conformation compatible with the P structure. (*d*) AP sheets form very quickly in the accelerated simulation systems, preventing formation of the P sheet by absorbing free monomers (the example trace for ILQINS is shown). (*e*–*g*) Representative AP single *β*-sheets formed in these simulations are shown. Side-chain orientation in the AP sheets initially formed is not regular; the structures (*e*–*g*) are mixed between the intrasheet orderings of classes 5 and 6 and of 7 and 8. To see this figure in color, go online.

To further investigate the early aggregation process, confirming the presence of AP *β*-sheet and testing for sequence dependence, three simulation systems of monomeric peptides (ILQINS, IFQINS, and TFQINS) in a continuum water model were constructed. An HREMD algorithm was implemented. Each system, each of 64 replicas, contained 64 peptides confined to a spherical volume of 50-Å radius, giving an effective concentration of 0.2 M. One replica was subjected to the unmodified AMBER Hamiltonian, whereas the remaining 63 replicas were subjected to increasingly strong perturbations intended to accelerate aggregation by confining peptides to approach a reference extended structure and to lie parallel to the *xy* plane. In replica exchange MD algorithms, conformations are exchanged between replicas such that the equilibrium conformational distribution for each replica is in theory preserved but is reached more quickly with the benefit of information from the other replicas (see Materials and Methods). With only 64 peptides, the full aggregation process cannot be investigated; however, the accelerated conformational search via this method allows initial steps to be probed. The majority of aggregates formed in each of the HREMD calculations had AP alignment of peptide strands, which is consistent with the SAXS/WAXS (Fig. 1), with the *ΔG* calculations (below), and with a qualitative consideration of the terminus-terminus interactions: each peptide has an unsatisfied charge at each end, favoring AP alignment. Fig. 3, *a*–*c* show the extended reference conformations, Fig. 3 *d* shows convergence for an example system (ILQINS), and Fig. 3, *e*–*g* show representative single *β*-sheets for each system taken from the simulations at their endpoints. Convergence is indicated by the number of P or AP hydrogen bonds formed: for a single giant *β*-sheet of all peptides in the system, a value of ∼180 hydrogen bonds would be expected (even inside a sheet, thermal excitation prevents all bonds from being continuously filled). The endpoints of the simulations, having ∼120 AP hydrogen bonds plus ∼5 P hydrogen bonds, in isolated *β*-sheets rather than steric zippers, are considered to be arrested states that could potentially require orders of magnitude more simulation time to progress to later stages of assembly (such as formation of two-sheet steric zippers) because of the much slower diffusion of sheets compared to monomeric peptides.

**Figure 4:**
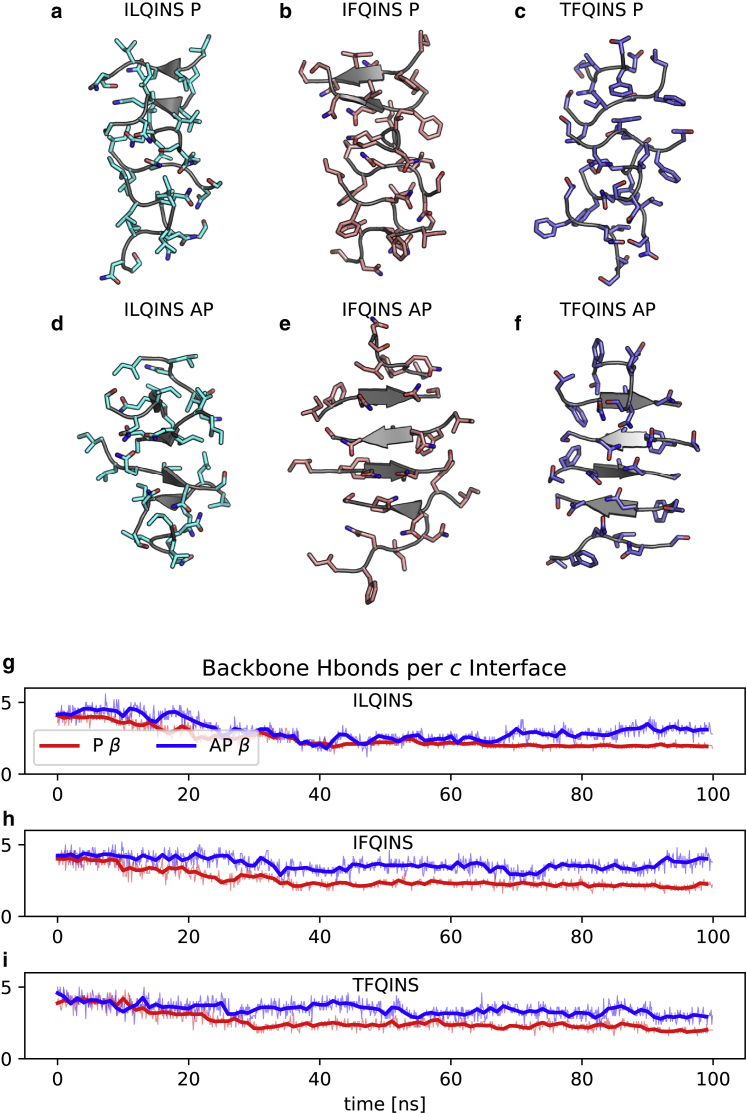
MD. Classical MD over 100 ns in explicit water shows that AP single *β*-sheets have greater configurational stability than P. (*a*–*f*) Shown are the P and AP *β*-structures consistent with classes 1 or 2 (P) and 5 or 6 (AP) at 100 ns for the three sequences studied. (*g*–*i*) The backbone hydrogen bonding declines more rapidly for the P structure in each case. Thick lines indicate a moving average over a 1-ns window. To see this figure in color, go online.

We confirm the stability of the AP aggregates by implementing classical MD simulations up to 100 ns in explicit water at 300 K and 1 atm, finding greater configurational stability for the AP rather than P single *β*-sheets. Final structures from these simulations are shown in Fig. 4.

### Thermodynamic analysis and sequence dependence

As a quantitative thermodynamic test of the idea that AP structures should lead to aggregation even though P structures are more common as insoluble fibrils or crystals, we evaluate binding free energy gains to join AP and P *β*-sheets in the *c* axis and at the steric zipper interface. This is done by calculating the difference between free energies of joined sheets of peptides (for example, two four-peptide sheets making an eight-peptide sheet) and separated sheets (for example, the same two four-peptide sheets no longer in contact), e.g.,

$$\text{Δ}G_{c}^{\circ }=\left[G_{118}-\left(G_{114}+G_{114}\right)\right],$$

where integer subscript triplets are the number of peptides in each dimension of a rectangular nanocrystal. Reference block-free energies *G*_*ijk*_ are calculated as averages over not less than 50 blocks sampled from the converged part (the 10th nanosecond) of the MD simulation. After a block is “cut” from the simulation system, its energy is minimized in a continuum solvent (58) so that the final free energy accounts for the electrostatics of solvent exposure and also contains at least some of the appropriate physical entropy change from creating an interface, particularly that related to ordering of the solvent.

Table 1 shows the interface formation energy compared for class 1 and class 5 structures. The larger free energy gain in *c* to stabilize AP structures is consistent with the tendency to see single-sheet AP oligomers more than P, whereas the stronger steric zipper formation energy ($\Delta G_{zip}^{\circ }$) for P structures is consistent with the eventual dominance of P-*β* crystals or of P multisheet fibrils. Interestingly, the pattern becomes more pronounced in line with increasing amyloidogenicity (14) of the sequence (TFQINS is a disease-associated mutation of IFQINS, the human wild-type sequence, which, in its turn, is more amyloidogenic in vitro than the wild-type chicken sequence ILQINS).

**Table 1:** Thermodynamics

		$\Delta G_{c}^{o}$	$\Delta G_{zip}^{o}$
ILQINS	class 1 (P)	−28.1 (2)	−18.6 (1)
	class 5 (AP)	−28.9 (2)	−16.3 (2)

*ΔΔG*:	1–5:	+0.8	−2.3

IFQINS	class 1 (P)	−27.1 (1)	−20.5 (1)
	class 5 (AP)	−29.3 (2)	−16.4 (3)

*ΔΔG*:	1–5:	+2.2	−4.1

TFQINS	class 1 (P)	−26.1 (1)	−21.7 (2)
	class 5 (AP)	−29.5 (2)	−13.1 (2)

*ΔΔG*:	1–5:	+3.4	−8.6

Standard binding free energy gain to construct a buried interface in the direction of the *β*-sheet hydrogen-bonding axis ($\Delta G_{c}^{o}$) and the side-chain steric zipper interface ($\Delta G_{zip}^{o}$). Units are kcal mol^−1^ per peptide buried by the interface. AP is stronger (*ΔΔG* > 0) in terms of sheet elongation but is weaker (*ΔΔG* < 0) in zipper stability. Increasing in vitro amyloidogenicity for the sequence IL < IF < TF is matched by the strengthening of this trend in the *ΔΔG*-values.

## Discussion

We have analyzed the aggregation of a set of amyloidogenic lysozyme-derived peptides. Even though mature structures deposited on surfaces have previously been shown using electron diffraction to be parallel *β*−sheets (14), solution SAXS/WAXS shows that antiparallel arrangement of *β*-strands is present together with the parallel. Accelerated MD was used to search the conformational space for small assemblies (not more than 64 peptides), finding that the peptides ILQINS, IFQINS, and TFQINS all formed single-sheet antiparallel structures that were stable on longer timescales (on the order of 100 ns) than the equivalent parallel structures. This observation is consistent with growing evidence from the wider literature that antiparallel *β*-structured oligomers precede parallel aggregates for many amyloid systems (27, 28, 29, 30, 31, 32, 33).

The idea that antiparallel should in general lead parallel is physically motivated by the stronger electrostatic interaction of two extended peptide strands in an antiparallel rather than parallel arrangement. This competes with the countervailing effect (observed for these systems and probably very common) that parallel *β*-sheet, with its smoother side surface (64), should have more favorable side chain-side chain stacking in the lateral phase of assembly. The formation of a stable lateral steric zipper is a crucial stage in amyloid self-assembly because the doubled thickness therefore roughly doubles the energetic cost to break the growing aggregate perpendicular to the *c* axis, therefore squaring the timescale for which it can be expected to endure in solution.

We expect the P-follows-AP effect to be quite general to amyloid assembly, particularly for small peptides for which the relative importance of the termini is larger, but evidence also supports this effect for the much larger A*β* (30, 31, 32) and *α*-synuclein peptides (33). For longer peptides, whatever other dipoles parallel to the strand axis are present (as well as effects due to the termini), they cannot prefer a parallel in-register alignment because such a structure necessarily stacks like charges with like. Counterexamples to the discussed phenomenon do exist; for example, similar accelerated MD simulations to those documented here have found that parallel *β*-dimerization is the initial step for the human islet amyloid polypeptide (65). This exception manifests what is a quite common pattern in amyloid formation, of each chain making a strand-turn-strand “horseshoe” motif such that the interface labeled *zip* in this work (strong for P-*β*) is formed at the same time as, or before, the interface labeled *c*. In the case that axial and zipper ordering are cooperative or that zipper ordering precedes axial ordering, the arguments presented here are not expected to apply; studies of the A*β* 17-42 (or “P3”) peptide, which forms such a horseshoe leading to cooperative sheet and zipper formation, have shown coexistence of P and AP, but with no implication that AP would be formed first (66).

This work does not discuss the exact kinetic of evolution from AP to P aggregates, presenting only thermodynamic information in relation to larger systems. Simulations have indicated that a group of six A*β*_40_ peptides can convert from AP to P without fully separating over some hundreds of microseconds (32); however, in larger systems, dissolution and reformation or secondary nucleation (67) of P by AP also enter as mechanisms for this evolution.

## Conclusion

Many amyloid aggregation processes have been understood to date as defined by parallel *β*-sheet. The observation that antiparallel aggregates should often dominate at the small length scales and timescales associated with toxic oligomers even when parallel structure is the ultimate fate demands a substantial re-examination of amyloid molecular medicine.

## Supporting Material

Document S1. Supporting Materials and Methods and Figs. S1–S2

Document S2. Article plus Supporting Material
