# Supplementary material for: Amyloid Evolution: Antiparallel Replaced by Parallel
Source: Biophys J. 2020 Apr 7;118(10):2526–36. doi: 10.1016/j.bpj.2020.03.023 (PMC7231890; doi:10.1016/j.bpj.2020.03.023)
Supplement: Document S1. Supporting Materials and Methods and Figs. S1–S2 [file mmc1.pdf]

Biophysical Journal, Volume *118*

Supplemental Information

Amyloid Evolution: Antiparallel Replaced by Parallel

Ali Asghar Hakami Zanjani, Nicholas P. Reynolds, Afang Zhang, Tanja Schilling, Raffaele Mezzenga, and Joshua T. Berryman

Supplementary material for:

"**Amyloid Evolution: Antiparallel replaced by Parallel**", Hakami-Zanjani, Reynolds, Zhang, Schilling, Mezzenga and Berryman. *Biophysical Journal* 2020.

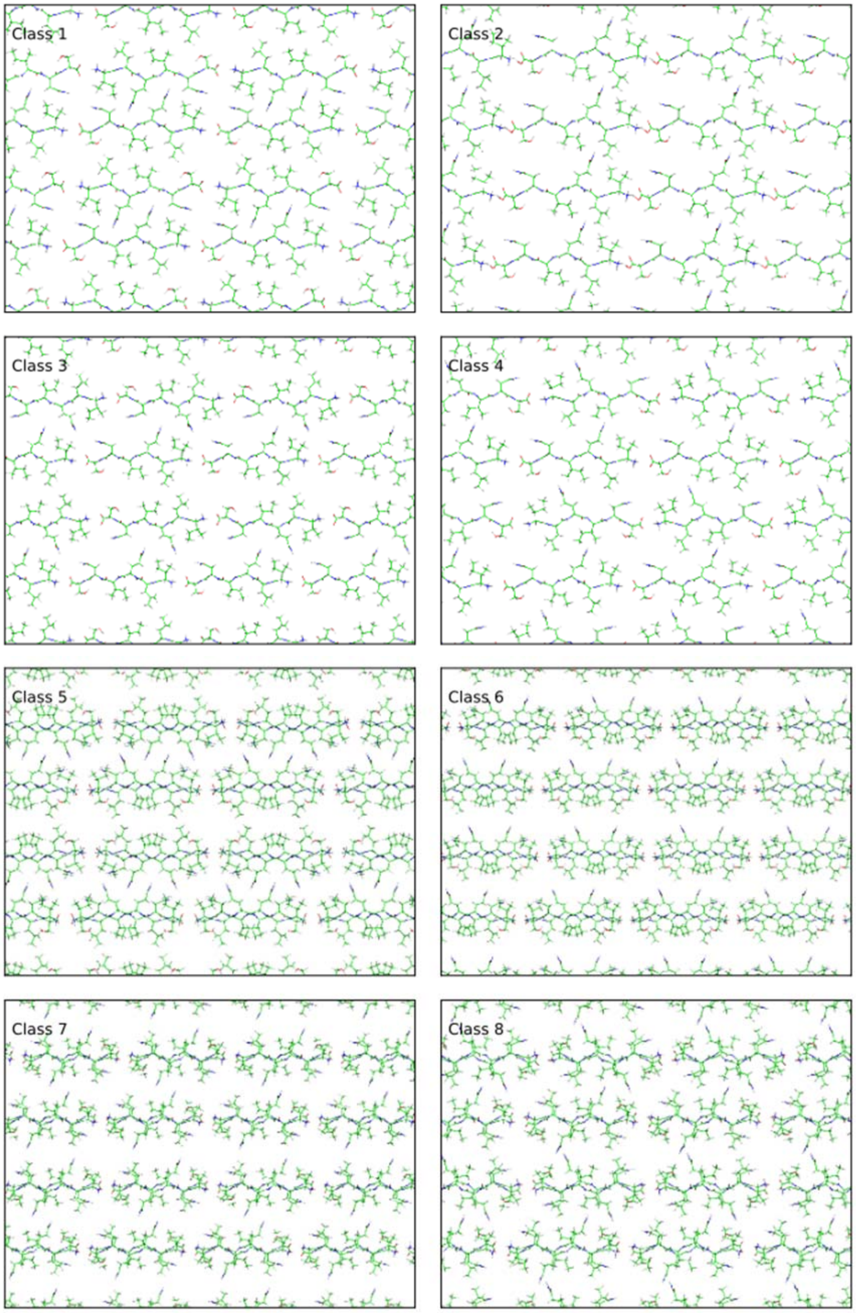

S5: Eight initial structures before MD simulations corresponding to the symmetry diagrams in Figure 1. View is an orthoscopic projection along the *c* (hydrogen-bonding) crystal axis, such that the P-beta structures 1-4 appear to be a 2D wallpaper.

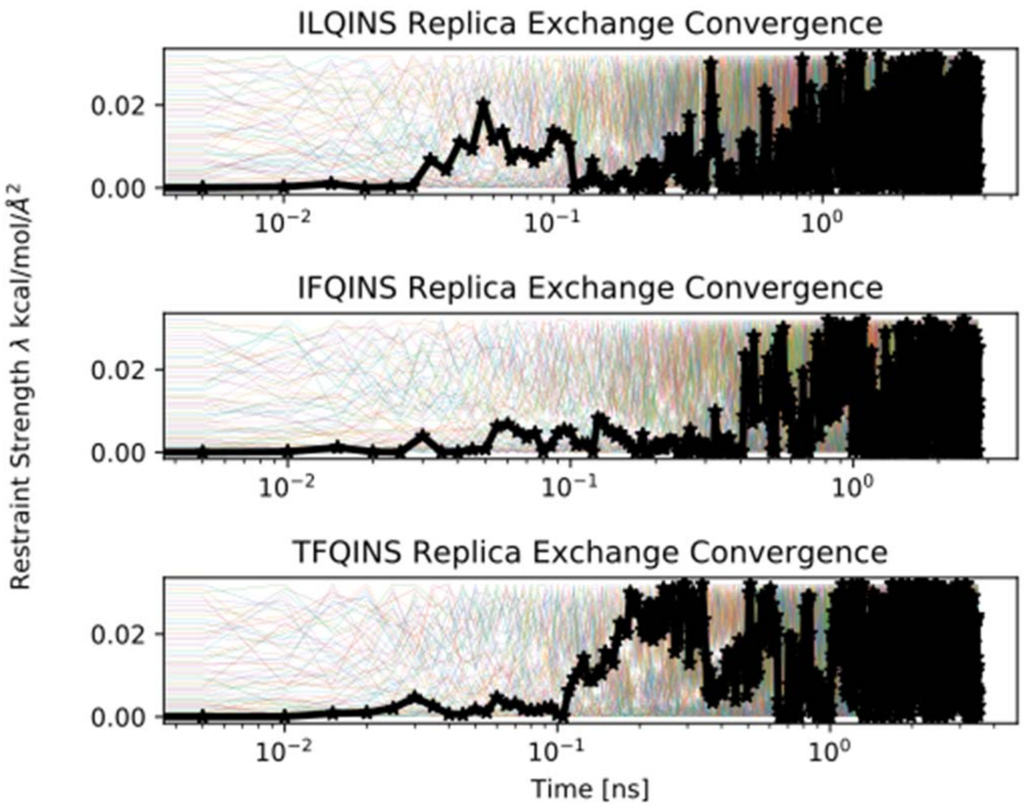

S6: Mixing of REMD trajectories. The black trace is chosen as an example to demonstrate that trajectories make the full tour of the replica set multiple times.

**Data Collection Parameters**	2014 Data	2019 Data
Instrument	SAXS/WAXS Beamline @ Australian Synchrotron with Pilatus 1M detector	SAXS/WAXS Beamline @ Australian Synchrotron with Pilatus 1M detector
Beam Geometry (µm)	300 x 200	250 x 500
Wavelength (Å)	1.033	1.033
q range ($Å^{-1}$)	0.03-0.9	0.03-1.8
Exposure Time (s)	1	1
Frames Collected	15	15
Concentration (mM)	1.5	1.5
Temperature (K)	298	298
**Structural Parameters**		
Cross-Sectional Radius of Gyration (R_c) (Å) (from SI(s) vs S^2 plot)	19.74 (Max S.Rg = 1.97) (Polydisperse sample?) (Data Unreliable)	24.8 (Max S.Rg = 2.08) (Polydisperse sample?) (Data Unreliable)
Radius of fibril R=R_c √2 (Å)	27.92	36.34
Forward Scattering (I_o) (Å)	0.00038	0.0006
**Software employed**		
Primary data reduction	Scatterbrain	Scatterbrain
Data processing	Scatterbrain/Primus	Scatterbrain
Ab initio analysis	N/A	N/A
Validation and averaging	Scatterbrain	Scatterbrain
Rigid-body modelling	N/A	N/A
Computation of model intensities	N/A	N/A
Graphics representations	Scatterbrain/Origin Pro 2019	Scatterbrain/Origin Pro 2019
**SASBDB Accession:**	SASDHQ5	SASDHK8

To obtain cross sectional radius of gyration R_c we plot SI(s) vs s^2 and (as for Guinier analysis) from the linear portion of the slope at low q we can estimate Rc.

$$sI(s) = \frac{m}{l} exp(-2\pi^2 R_c^2 s^2)$$
